# Skin check partner assistance for melanoma skin self-examination by at-risk patients: it takes two to identify melanomas

**DOI:** 10.2217/fon-2020-0265

**Published:** 2020-04-15

**Authors:** June K Robinson

**Affiliations:** 1 Department of Dermatology, Northwestern University Feinberg School of Medicine, Chicago, Illinois 60611, USA

**Keywords:** early detection, melanoma, skin self-examination

In the USA, there will be an estimated 100,350 new cases of melanoma and 6850 deaths in 2020 [1]. By 2030, the number of newly diagnosed cases is expected to be more than double and the annual cost of treating newly diagnosed melanomas is estimated to triple from $457 million in 2011 to $1.6 billion in 2030 [2–4]. Early detection determines the disease stage, prognosis, treatment and cost to the payer [5]. Time to presentation for care is a key determinant of patients’ outcome. Compared with other cancers, melanoma has the longest delays measured as the median time from symptom onset to patient presentation [6]. Since most melanomas are visible on the surface of the skin at a curable phase in their evolution, people at-risk to develop melanoma can check their moles by performing skin self-examination (SSE). In the general community, the annual prevalence of self-reported SSE, defined as any area of skin checked sporadically, ranges from 8 to 21% [7]. Women self-detect more than half of melanomas and melanomas detected by women had a better prognosis than those detected by men because they were identified at an earlier stage [8,9].

Waiting times for appointments with dermatologists, dermatologist shortages in rural areas, out of pocket costs and distance to the nearest dermatologist may discourage people from receiving dermatological care. A health disparity exists in the USA for residents of rural counties. Rural counties in the USA have higher incidence rates for melanoma than metropolitan counties and higher mortality rates [10]. The higher incidence is most likely due to unprotected sun exposure in a predominantly non-Hispanic white population engaged in agricultural and other outdoor occupations. Rural residents, who are older, more socioeconomically disadvantaged and less educated than their urban counterparts, have greater travel distances and transportation difficulties in reaching healthcare providers (HCPs) for both general and specialty care than urban residents [11]. Until self-management for early detection of melanoma among at-risk people and health disparities are effectively addressed, patients will present late in the disease incurring significant mortality, morbidity and healthcare costs.

In 2018, the US Preventive Services Task Force concluded that current evidence is insufficient to assess the balance of benefits and harms of SSE to prevent skin cancer [12]. This US Preventive Services Task Force recommendation does not address SSE among people at-risk to develop melanoma. Organizations defined individuals at-risk of developing melanoma as people with:

- Skin type Fitzpatrick scale I–III (skin that sustains a sunburn);
- A personal or family history of melanoma;
- A personal history of nonmelanoma skin cancer (basal or squamous cell carcinoma);
- A history of multiple sunburns;
- A history of 10 or more indoor tanning sessions in a lifetime;
- Having multiple atypical nevi and/or;
- Having ongoing immuocompromise [13].

The American Academy of Dermatology encourages self-advocacy through routine SSE and regular skin cancer screening by dermatologists for at-risk patients, who require routine physician screening [14]. Physician endorsement of SSE to at-risk patients and access to SSE skills training are needed to help patients self-manage early detection of melanoma. Among those at-risk to develop melanoma, melanoma survivors may be reached by physicians because patients receive routine follow-up care from physicians every 3–12 months [13], While the importance of early detection is usually recognized by melanoma survivors, they may not realize that 7–12% of patients with a history of melanoma develop a second primary melanoma [15]. Thus, physicians need to inform melanoma survivors of their risk of developing another melanoma. Since a new melanoma may arise in the interval between physician follow-up visits, physician endorsement of SSE is worthwhile. The benefit of SSE was demonstrated in a 20 year follow-up case control study of people newly diagnosed with melanoma in 1987–89 [16]. In this study, skin awareness prompted by SSE was associated independently with decreased risk of melanoma death (HR: 0.46; 95% CI: 0.28–0.75; p < 0.01) [16].

In our randomized clinical trial, SSE skills training for at-risk melanoma survivors and their skin check partners enabled pairs to accurately assess moles and track concerning moles for change [17,18]. This evidence–based systematic assessment of moles on most areas of the skin, including scalp, back, buttocks and feet was taught and performed by melanoma survivors and their skin check partners. Skills training was provided in a workbook with color illustrations that taught the pair to score the following three features of a mole using a scale of 1-3 (1 = normal, 2 = unsure, 3 = not normal):

- border (smooth = 1 or jagged = 3);
- color (one-two colors = 1 or a variety of colors = 3) and;
- diameter of a mole (<4 mm = 1, 5 mm = 2, or >6 mm = 3).

The pair was encouraged to start by checking a mole that they both could easily see, for example, the forearm. Then, the pair worked together using a mm ruler to measure the widest part of the mole and a lighted magnifying lens to see the border and color of the mole. Pairs discussed the scores they gave to each feature and agreed upon the score for border, color, and diameter before entering the scores in a diary. The decision about seeking healthcare for a concerning mole was based upon the sum of the features as follows:

• 3 = benign, stop checking the mole;
• 4–7 = check the mole in one month;
• 8–9 = make an appointment with the HCP to have the mole checked in about 2–3 weeks.

The recommendation to have a mole with a score of 8–9 checked by the HCP in 2–3 weeks was supported by study physicians seeing patients within 2–3 weeks. Acceptance of SSE by melanoma survivors in our study may have been enabled by the assurance of ready access to study physicians if a concerning mole was detected. SSE was an adjunct to HCP examination. The patients’ diary documenting change in a mole assisted HCPs in deciding to perform a skin biopsy. Partner-assisted SSE and subsequent clinical presentation to the HCP for concerning moles rely on self-management, adoption of decision rules and taking appropriate action.

The skin check partner served three important functions: the partner saw areas of the body that the at-risk person cannot easily see themselves (ears, back of neck, scalp); reinforced the need to do SSE by reminding the melanoma survivor to do SSE or scheduling times to do partner assisted SSE and helped to build confidence in making decisions about scoring the features of the mole and seeking an appointment with the HCP. Each pair developed their own way of doing SSE. For some, the melanoma survivor checked all the places he or she was able to see alone and invited the partner to check places that were hard to see. Others did the total body skin check together. Pairs were encouraged to reward themselves after completing SSE by doing something they would enjoy doing together, for example going to a movie. The case example of a wife of a 65-year-old man, who was a survivor of stage II A melanoma, illustrates the benefit of partner assisted SSE. The wife invited her husband, the melanoma survivor, to sit down so she could see the vertex of his balding scalp. She found a suspicious looking dark spot and started to score the border, color and diameter. As she assessed each feature, she told him the score. He placed the score in his diary. The border was jagged (score 3), the diameter was 7 mm (score 3), and she could not decide about the color (score = 2). The total score was 8. She and her husband decided they needed to have the HCP check the concerning mole on the scalp. In this example, the partner’s help in identifying this concerning mole of the scalp was especially important because patients with melanoma of the scalp died at 1.84-times (HR: 1.84; 95% CI: 1.62–2.10) the rate of those with melanoma in any other location [19]. The reason for the increased mortality of scalp melanoma may be presentation for care at a more advanced stage than other locations, which are easier for the patient to see. In this case, for example, the concerning mole of the scalp was biopsied and was a stage IA melanoma. Further support for the role of the partner came from a study showing that there was increased melanoma mortality among bereaved melanoma survivors, whose partners died [20]. This may be partly explained by delayed detection resulting from the loss of a partner who checks difficult to see areas of the body.

Common patient burdens such as HCP appointment scheduling difficulties, time away from work and family, transportation constraints and the cost of the physician visit may be reduced by SSE training. Lack of confidence in SSE skills may cause some people to doubt their self-diagnosis and fail to make an appointment with the HCP or follow a mole for change. Physicians may boost patients’ SSE confidence by saying, “You are doing really well checking moles”. Another form of encouragement that physicians may offer is the advice that asking a relative to check places that are hard to see would be helpful. People may find SSE too much of ‘a bother’, cease monthly SSE, and perform SSE at 2–3-monthly intervals. It is best if physicians recognize the difficulty of monthly SSE, which was intended to form a sustainable habit and support SSE at the less frequent interval of every 2–3 months, which in our research was sufficient to detect change in a concerning mole without having disease detected at an advanced stage. In times of restricted access to healthcare, such as during the COVID-19 pandemic, people at-risk of developing melanoma may benefit from having the skills to self-manage early detection of melanoma. Self-management may reduce patient demand for physician screening of benign moles and improve access to physician screening for concerning moles.
